# Investigating the prevalence of primary thyroid dysfunction in obese and overweight individuals: Tehran thyroid study

**DOI:** 10.1186/s12902-021-00743-4

**Published:** 2021-04-30

**Authors:** Mahdi Mahdavi, Atieh Amouzegar, Ladan Mehran, Elham Madreseh, Maryam Tohidi, Fereidoun Azizi

**Affiliations:** 1 Endocrine Research Center, Research Institute for Endocrine Sciences, Shahid Beheshti University of Medical Sciences, P.O. Box: 19395-4763, Tehran, Iran; 2 Institute of Medical Science and Technology (IMSAT), Shahid Beheshti University, Tehran, Iran; 3 Department of Epidemiology and Biostatistics, School of Public Health, Tehran University of Medical Sciences, Tehran, Iran; 4 Prevention of Metabolic Disorders Research Center, Research Institute for Endocrine Sciences, Shahid Beheshti University of Medical Sciences, Tehran, Iran

**Keywords:** Obesity, Overweight, Thyroid dysfunction, Tehran thyroid study, Hypothyroidism

## Abstract

**Background:**

Due to the increasing worldwide prevalence of obesity, it is essential to determine the prevalence of obesity-related thyroid dysfunctions. The purpose of this study was to investigate the prevalence of thyroid dysfunctions, namely hypothyroidism and hyperthyroidism, and their association with BMI among adult Iranian overweight and obese individuals.

**Method:**

This cross-sectional study was carried out within the framework of the Tehran Thyroid Study (TTS); 5353 participants (57.5% female) entered our study. Anthropometric measurements were performed. Serum levels of thyroid-stimulating hormone (TSH), free thyroxine (FT4), and thyroid peroxidase antibody (TPOAb) were assayed. We categorized individuals into 3 BMI groups (normal-weight, overweight and obese), then calculated prevalence rate, odds ratio (OR), and 95% confidence interval (CI) for outcomes in overweight and obese groups. The normal-weight group was used as the control group.

**Results:**

We found a higher prevalence of hypothyroidism (11.6% vs 8.2% Total, 4.0% vs 1.1% overt and 7.6% vs 7.1% subclinical, *P* < 0.001) and TPOAb positivity (17.3% vs 11.6%, *P <* 0.001) in obese participants compared with normal-weight participants. Hyperthyroidism’s overall prevalence was 4.2, 5.7, and 4.9% in obese, overweight, and normal-weight groups, respectively. Obesity was associated with higher odds of overt hypothyroidism (OR: 2.0, 95% CI: 1.15–3.49, *P* < 0.05) and TPOAb positivity (OR: 1.29, 95% CI: 1.04–1.60, *P <* 0.05) after adjusting for confounding variables. In contrast, no association was observed between the overweight group and the odds of hypothyroidism and TPOAb positivity in the adjusted results.

**Conclusions:**

Obesity was associated with an increased risk of overt hypothyroidism and TPOAb positivity.

## Background

Obesity and overweight are major risk factors for disability-adjusted life-years (DALY) and mortality; obesity was linked to 4 million deaths and 120 million DALYs, 7.1 and 4.9% of all global deaths and DALYs, in 2015. The prevalence of obesity has drastically increased in the past three decades around the world. The global prevalence of obesity in adults was estimated to be 12%, with a higher prevalence rate in women than men [1, 2]. Obesity has become a significant public health concern in many countries, including Iran; the prevalence of obesity and overweight among the Iranian population above 18 has been estimated to be around 21 and 39%, respectively [3, 4].

Thyroid dysfunctions, after diabetes, are the second most prevalent metabolic disorders in the world. Many elements, including biological and geographical factors, affect thyroid dysfunction prevalence rates among various world regions. Among the Iranian population, a prevalence of 0.69% for overt hyperthyroidism and 1.52% for subclinical hyperthyroidism have been reported [5]. Moreover, the prevalence of overt and subclinical hypothyroidism in Iran is estimated to be 2.0 and 5.5%, respectively [6].

Obesity is accompanied by various physiological and pathological changes in body organs, including the thyroid gland [7]. Many studies have investigated the relationship between obesity and thyroid dysfunction, but the results of these studies are still inconsistent. Most studies have shown that body mass index (BMI) is positively associated with serum thyrotropin (TSH) and the prevalence of hypothyroidism in obese individuals [8–12]. However, results regarding the relation between BMI and serum-free thyroid hormone levels are controversial. While several studies have reported that BMI is negatively associated with free thyroxine (FT4), others have found positive or no association between BMI and FT4 [10, 13–16].

With the increasing frequency of obesity, determining the prevalence of obesity-related thyroid dysfunctions is essential for designing precise community-based health guidelines. To the best of our knowledge, few studies have explored the prevalence of thyroid dysfunctions among obese individuals in a Middle Eastern population. The aim of this population-based, cross-sectional study was to investigate the prevalence of thyroid dysfunctions, namely hypothyroidism and hyperthyroidism, and their association with BMI among overweight and obese individuals in an adult Iranian population.

## Materials and methods

### Study setting

The present study was carried out within the framework of the Tehran Thyroid Study (TTS) retrospective cohort study in the Iranian city of Tehran, which is considered an iodine sufficient region. The TTS study was conducted as a part of the Tehran Lipid and Glucose Study (TLGS), which is a long-term ongoing community-based study for the identification and prevention of non-communicable disorders among the Iranian population living in district No.13 (population toll: 276,027, Iran National Census, 2010) located in the eastern parts of Tehran; this district is under the coverage of Shahid Beheshti University of Medical Sciences and Health Services. The population of the above-mentioned area represents the overall population of Tehran (Iran National Census, 1996) [17].

### Study population

Fifteen thousand five (15,005) individuals aged ≥3 years were selected to participate in the TLGS study using multistage stratified cluster sampling. Baseline measurements were documented, and every 3 years, participants were invited for follow-up assessments. From 10,368 TLGS participants aged ≥20 y., 5783 individuals who had serum samples for measuring thyroid function tests at baseline (February 1999–August 2001) were randomly selected Between March 1997 and December 2004 to participate in the TTS study.

**Fig. 1:**
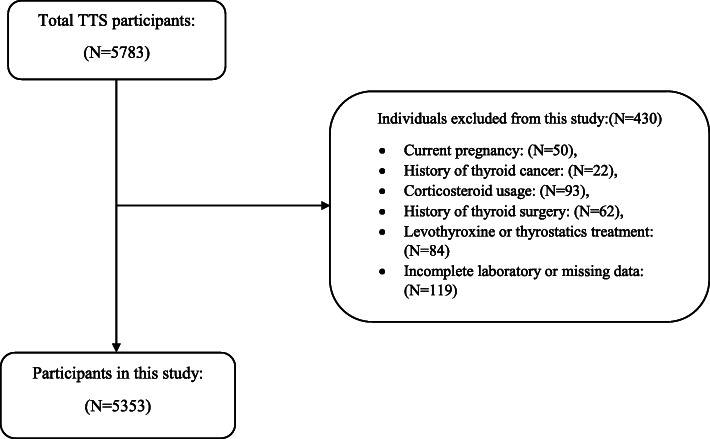
Flowchart of the study population sampling

In this study, all participants from the baseline TTS study were included. Exclusion criteria (Fig. 1) for this study were a history of thyroid cancer (n: 22), a history of thyroid surgery (n: 62), current pregnancy (n: 50), corticosteroid usage (n: 93), levothyroxine or thyrostatics treatment (*n* = 84), and having incomplete laboratory or missing data (n: 119). As a result, 430 individuals were excluded, and 5353 participants remained in our study.

### Clinical evaluation

All participants were referred to health centers for clinical evaluations; trained physicians performed clinical evaluations in every step of this study. Participants were surveyed to obtain information regarding past medical history, current medication, radioiodine intake, and smoking habits. Anthropometric measurements were taken without shoes with participants wearing light clothing; weight and height were measured using a digital electronic weighing scale (Seca 707; range 0.1–150 kg; Seca, Hanover, MD) with an accuracy of up to 100 g (checked regularly for precision every ten measurements) and a tape meter stadiometer respectively. Waist circumference (WC) was measured at the narrowest level over light clothing, using an upstretched tape meter, without any pressure to the body surface. The measured values were rounded to have a precision of 1 cm. Body mass index (BMI) was calculated by dividing the weight in kilograms by the square of height in meters.

### Laboratory measurements

Study participants were asked to fast overnight to ensure accurate lab results. Blood samples were collected between 7:00 AM and 9:00 AM after 12 to 14 h of overnight fasting. The electrochemiluminescence immunoassay (ECLIA) method was used on − 70 °C stored serum samples to measure TSH and FT4 using Roche Diagnostics kits & Roche/Hitachi Cobas e-411 analyzer (GmbH, Mannheim, Germany). Lyophilized quality control substance (Lyphochek Immunoassay plus Control, Bio-Rad Laboratories) was utilized to assess the assay’s accuracy. The intra- and interassay Coefficients of Variability (CVs)were 1.5 and 4.5% for TSH and 1.3 and 3.7% for FT4 determinations, respectively. Thyroid peroxidase antibody (TPOAb) was measured by the immunoenzymometric assay (IEMA), using an appropriate kit (Monobind, Costa Mesa, CA, USA) and the Sunrise ELISA reader (Tecan Co., Salzburg, Austria); inter- and intra-assay CVs were 3.9 and 4.7%, respectively. All lab analysis procedures were performed at the research laboratory of the Research Institute for Endocrine Sciences (RIES).

### Definition of variables

We categorized individuals into three groups based on their BMI; participants with BMI ranging from 18.5 kg/m^2^ to 24.9 kg/m^2^ were classified as normal-weight (normal BMI), individuals with BMI between 25 kg/m^2^ to 29.9 kg/m^2^ and equal or greater than 30 kg/m^2^ were classified as overweight and obese, respectively. Furthermore, we also defined sex-specific female and male BMI groups to separately investigate the effects of obesity on the thyroid function of each gender. Euthyroidism was defined as serum TSH and FT4 within the normal reference range of the study population (TSH: 0.32–5.06 mIU/L; FT4: 0.91–1.55 ng/dL); furthermore, we divided thyroid dysfunction as outcome into 4 categories: 1) subclinical hyperthyroidism (defined as TSH < 0.32 and 0.91 ≤ FT4 ≤ 1.55) 2) overt hyperthyroidism (defined as TSH < 0.32 and FT4 > 1.55) 3) subclinical hypothyroidism (defined as TSH > 5.06 and 0.91 ≤ FT4 ≤ 1.55) 4) overt hypothyroidism (defined as TSH > 5.06 and FT4 < 0.91); we applied population-specific TSH and FT4 reference ranges based on results obtained from a previous study on TTS participants. Regarding TPOAb, we used an estimated cutoff value of 32.8 IU/mL in males and 35.0 IU/mL in females [18]. We classified individuals with serum TPOAb levels above the cutoff values as TPOAb positive. A value of 90 cm, obtained from previous studies on the Iranian population, was used as the cutoff point to denote abnormal WC in both men and women [19].

Regarding the smoking status, participants were divided into ever smoker and never smoker groups. Ever smoker was defined as an individual who has smoked 100 cigarettes in his or her lifetime and is also currently smoking cigarettes, regularly or irregularly. A never-smoker individual was defined as a person who has never smoked or smoked less than 100 cigarettes in his or her lifetime.

### Data analysis

Normally distributed variables were expressed as mean ± SD. Furthermore, continuous variables with skewed distribution were expressed as median with IQR, and categorical variables were presented as frequencies (%). Kruskal-Wallis Test and chi-square tests were used to compare continuous and categorical variables, respectively.

Since in our modeling more than two thyroid function states as outcomes were present, multinomial logistic regression was utilized to model our data and subsequently calculate odds ratios (OR) and 95% confidence intervals (CI) for different outcome groups. Additionally, we used normal-weight participants as the control group to calculate OR in our logistic models. Since TPOAb positivity had a binary outcome, positive or negative, we used binary logistic regression to model and calculate OR and 95% CI for TPOAb positivity. To further assess the impact of obesity on thyroid disorders, we utilized Spearman’s rank correlation coefficient and linear regression modeling to analyze the correlation and association between thyroid parameters (TSH, FT4, and TPOAb level) and BMI. To simplify the interpretation of results from our linear models, natural log transformation was used for parameters that had skewed distribution. As a result, the percent of changes shown in Table 3 are expressed as 100*(exp (coefficient)-1).

We classified age, sex, TPOAb positivity, and smoking as confounding factors for calculating adjusted outcomes from our regression analysis. All statistical analyses were performed by SPSS 23.0 software.

### Ethics statement

The protocol for the TTS study was approved by the Ethics Committee of the Research Institute for Endocrine Sciences (RIES) of Shahid Beheshti University of Medical Sciences; the study was conducted in accordance with the Declaration of Helsinki. Participants received ample information about the aim and steps of the TTS study. Afterward, all individuals willing to participate were asked to fill a written informed consent and were informed of their right to leave the study at any stage.

## Results

### Characteristics of the study population

A total number of 5353 individuals (42.5% male, 57.5% female) were included in this study. The mean age was 40 ± 14 (SD), 42 ± 15, and 39 ± 14 years for the total study population, male participants and female participants, respectively. The mean BMI of the study population was 26.5 ± 4.6 kg/m^2^. The prevalence of obesity was significantly higher among female participants than male individuals (26.5% vs. 14.5%, *P* < 0.001), and men had higher rates of overweight than women (42.0% vs. 38.8%, *P <* 0.001). Analysis of WC revealed the presence of abnormal WC in 44.6% of the study population (40.4% female, and 50.4% male). Median WC was 88 (17), 86 (18), and 90 (16) cm for the general, female, and male population, respectively (Table 1). Regarding smoking status, 792 participants (14.8%) had a positive, and 4561 (85.2%) had a negative history of smoking. Detailed clinical and biochemical characteristics of our study population are presented in Table 1.

**Table 1:** *Clinical and Biochemical Characteristics of the Study Population*

TOTAL	Normal BMI	Overweight (BMI ≥ 25)	Obese (BMI ≥ 30)	*p*-value^+^
(n, %)	(2057, 38.4%)	(2152, 40.2%)	(1144, 21.4%)	
Age (year)	36 ± 14	42 ± 14	45 ± 13	< 0.001
TSH (mU/L)	1.56 (1.68)	1.59 (1.66)	1.69 (1.81)	< 0.001
FT4 (ng/dL)	1.25 (0.24)	1.20 (0.24)	1.17 (0.24)	< 0.001
TPOAb (IU/ml)	5.43 (7.94)	5.60 (8.60)	6.34 (12.11)	< 0.001
Waist Circumference				< 0.001
WC	77.0 (11.0)	90 (10)	101 (9)	
Abnormal WC ratio	6.8%	55.5%	92.3%	
Smoking Status				< 0.001
Ever	358 (17.4%)	313 (14.5%)	121 (10.6%)	
Never	1699 (82.6%)	1839 (85.5%)	1023 (89.4%)	
TPOAb positivity				< 0.001
Yes	239 (11.6%) 1818	299 (13.9%)	198 (17.3%)	
NO	(88.4%)	1853 (86.1%)	946 (82.7%)	
Thyroid Status				< 0.001
Euthyroid	1787 (86.9%)	1837 (85.5%)	963 (84.2%)	
Subclinical hypothyroidism	146 (7.1%)	144 (6.7%)	87 (7.6%)	
overt hypothyroidism	23 (1.1%)	48 (2.2%)	46 (4.0%)	
Total hypothyroidism	169 (8.2%)	192 (8.8%)	133 (11.6%)	
Subclinical hyperthyroidism	57 (2.8%)	85 (3.9%)	31 (2.7%)	
overt hyperthyroidism	44 (2.1%)	38 (1.8%)	17 (1.5%)	
Total hyperthyroidism	101 (4.9%)	123 (5.7%)	48 (4.2%)	
FEMALE (3079, 57.5%)	**(1068, 34.7%)**	**(1196, 38.8%)**	**(815, 26.5%)**	
Age (year)	33 ± 12	41 ± 13	45 ± 12	< 0.001
TSH (mU/L)	1.78 (1.99)	1.87 (2.16)	1.85 (2.07)	0.497
FT4 (ng/dL)	1.20 (0.22)	1.15 (0.23)	1.14 (0.24)	< 0.001
TPOAb (IU/ml)	5.81 (10.52)	6.33 (12.64)	6.86 (17.25)	< 0.001
Waist Circumference				< 0.001
WC	75 (10)	87 (11)	100 (12)	
Abnormal WC ratio	3.9%	39.4%	89.7%	
Smoking Status				0.875
Ever smoker	36 (3.4%)	45 (3.8%)	30 (3.7%)	
Never smoker	1032 (96.6%)	1151 (96.2%)	785 (96.3%)	
TPOAb positivity				0.004
Yes	154 (14.4%)	209 (17.5%)	165 (20.2%)	
NO	914 (85.6%)	987 (82.5%)	650 (79.8%)	
Thyroid Status				0.008
Euthyroid	882 (82.6%)	971 (81.2%)	663 (81.3%)	
Subclinical hypothyroidism	110 (10.3%)	113 (9.4%)	78 (9.6%)	
Overt hypothyroidism	20 (1.9%)	39 (3.3%)	41 (5.0%)	
Total hypothyroidism	130 (12.2%)	152 (12.7%)	120 (14.6%)	
Subclinical hyperthyroidism	33 (3.1%)	51 (4.3%)	20 (2.5%)	
Overt hyperthyroidism	23 (2.2%)	22 (1.8%)	13 (1.6%)	
Total hyperthyroidism	56 (5.3%)	73 (6.1%)	33 (4.1%)	
MALE (2274, 42.5%)	**(989, 43.5%)**	**(956, 42.0%)**	**(329, 14.5%)**	
Age (year)	39 ± 16	44 ± 14	43 ± 14	< 0.001
TSH (mU/L)	1.41 (1.29)	1.38 (1.21)	1.46 (1.36)	0.570
FT4 (ng/dL)	1.29 (0.25)	1.25 (0.23)	1.24 (0.25)	< 0.001
TPOAb (IU/ml)	5.24 (6.45)	5.08 (5.67)	5.08 (7.80)	0.845
Waist Circumference				< 0.001
WC	80 (10)	94 (8)	104 (9)	
Abnormal WC ratio	9.8%	75.6%	98.8%	
Smoking Status				0.058
Ever smoker	322 (32.6%)	268 (28.0%)	91 (27.7%)	
Never smoker	667 (67.4%)	688 (72.0%)	238 (72.3%)	
TPOAb positivity				0.686
Yes	85 (8.6%)	90 (9.4%)	33 (10.0%)	
NO	904 (91.4%)	866 (90.6%)	296 (90.0%)	
Thyroid Status				0.275
Euthyroid	905 (91.5%)	866 (90.6%)	300 (91.2%)	
Subclinical hypothyroidism	36 (3.6%)	31 (3.2%)	9 (2.7%)	
Overt hypothyroidism	3 (0.3%)	9 (0.9%)	5 (1.5%)	
Total hypothyroidism	39 (3.9%)	40 (4.21%)	14 (4.3%)	
Subclinical hyperthyroidism	24 (2.4%)	34 (3.6%)	11 (3.3%)	
Overt hyperthyroidism	21 (2.1%)	16 (1.7%)	4 (1.2%)	
Total hyperthyroidism	45 (4.5%)	50 (5.3%)	15 (4.6%)	

*Normally distributed variables were expressed as mean ± SD, non-normally distributed variables as median with IQR, and categorical variables as numbers with percentages (%).*^*+*^ *Kruskal Wallis Test and chi-square test were used for intergroup comparison of Continuous and categorical variables, respectively. P-values less than 0.05 were considered statistically significant. Abnormal WC ratio displays the percentage of individuals within each BMI group with an abnormal waist circumference. BMI* body mass index, *TSH* thyroid-stimulating hormone, *TPOAb* thyroid peroxidase antibody, *FT4* free thyroxine, *WC* waist circumference

### Prevalence of thyroid dysfunction

The overall prevalence of hypothyroidism and hyperthyroidism in the study population was 9.2 and 5.1%, respectively. We found a significantly higher prevalence of hypothyroidism in obese individuals (11.6, 4.0% overt, and 7.6% subclinical) than individuals with normal BMI (8.2, 1.1% overt, and 7.1% subclinical). Regarding hyperthyroidism, the obese category showed a slightly lower prevalence of total hyperthyroidism than normal weight (4.2% vs. 4.9%, *P* < 0.001). In contrast, the overweight group displayed a higher prevalence of total hyperthyroidism than the normal weight category (5.7% vs. 4.9%, *P <* 0.001) (Table 1). Additionally, sex-specific results showed that the prevalence of overt hypothyroidism was noticeably higher among obese women than normal-weight ones (5.0% vs. 1.9, *P* < 0.05). Furthermore, the prevalence of total hyperthyroidism was lower in obese (4.1% vs. 5.3%, *P <* 0.05) and higher in overweight (6.1% vs. 5.3%, *P <* 0.05) female categories than the normal weight female group. In contrast, no statistically significant difference in the prevalence of hypothyroidism and hyperthyroidism was observed between different classes of BMI in men (Table 1).

Unadjusted logistic regression results showed that the odds of overt hypothyroidism were positively associated with obese (OR: 3.71, 95% CI: 2.24–6.16, *P* < 0.05) and overweight (OR: 2.03, 95% CI: 1.23–3.35, *P <* 0.05) BMI categories; this positive association was observed in obese (OR: 2.73, 95% CI: 1.58–4.70, *P <* 0.05) and overweight (OR: 1.77, 95% CI: 1.03–3.06, *P <* 0.05) female participants. Likewise, male overweight (OR: 3.14, 95%CI: 0.85–11.62, *P <* 0.05) and obese groups (OR: 5.03, 95% CI: 1.19–21.17, *P <* 0.05) also showed a statistically significant association with overt hypothyroidism in unadjusted results (Table 2). After adjusting the model for age, sex, TPOAb positivity, and smoking as confounding factors, only obese total (OR: 2.0, 95% CI: 1.15–3.49, *P <* 0.05), obese female (OR: 1.71, 95%CI: 0.94–3.11, *P <* 0.05) and obese male (OR: 4.55, 95% CI: 1.04–19.87, *P <* 0.05) groups had significant positive associations with the odds of overt hypothyroidism. We found no statistically significant association between subclinical hypothyroidism and BMI categories in our adjusted or unadjusted models (Table 2). We also observed a positive association between subclinical hyperthyroidism and the overweight group in our unadjusted results (OR: 1.45, 95% CI: 1.03–2.04, *P <* 0.05). However, this association was not observed in adjusted results or other BMI groups. Furthermore, no association was found between overt hyperthyroidism and BMI categories in adjusted or unadjusted results (Table 2).

**Table 2:** *Comparison of Odds Ratios of Thyroid Dysfunctions Among Different BMI Categories*

	Overt hypothyroidism^a^	Subclinical hypothyroidism^a^	Overt hyperthyroidism^a^	subclinical hyperthyroidism^a^	TPOAb positivity^b^	Overt hypothyroidism^a^	Subclinical hypothyroidism^a^	Overt hyperthyroidism^a^	subclinical hyperthyroidism^a^	TPOAb positivity^b^
	Crude OR (CI)					Adjusted^c^ OR (CI)				
**TOTAL**										
Over weight	2.03^*^ (1.23, 3.35)	0.96 (0.75,1.22)	0.84 (0.54,1.30)	1.45^*^ (1.03, 2.04)	1.23^*^ (1.02,1.47)	1.53 (0.91,2.60)	0.93 (0.72,1.21)	0.79 (0.50,1.25)	1.27 (0.89,1.79)	1.13 (0.94,1.36)
Obese	3.71^*^ (2.24,6.16)	1.11 (0.84,1.46)	0.72 (0.41, 1.26)	1.01 (0.65, 1.57)	1.59^*^ (1.30,1.95)	2.00^*^ (1.15,3.49)	0.90 (0.66,1.22)	0.62 (0.34,1.12)	0.81 (0.51,1.28)	1.29^*^ (1.04,1.60)
**FEMALE**										
Over weight	1.77^*^ (1.03,3.06)	0.94 (0.71,1.24)	0.87 (0.48,1.57)	1.40 (0.90, 2.20)	1.26^*^ (1.002,1.58)	1.29 (0.72,2.31)	0.93 (0.69,1.25)	0.77 (0.41,1.43)	1.23 (0.77,1.96)	1.18 (0.93,1.49)
Obese	2.73^*^ (1.58,4.70)	0.96 (0.71,1.30)	0.75 (0.38, 1.49)	0.81 (0.46, 1.42)	1.51^*^ (1.18,1.92)	1.71^*^ (0.94,3.11)	0.93 (0.66,1.30)	0.62 (0.29,1.29)	0.67 (0.37,1.21)	1.37^*^ (1.06,1.78)
**MALE**										
Over weight	3.14^*^ (0.85,11.62)	0.90 (0.55,1.47)	0.80 (0.41, 1.54)	1.48 (0.87,2.52)	1.11 (0.81,1.51)	2.94 (0.77,11.17)	0.97 (0.57,1.64)	0.81 (0.41,1.58)	1.33 (0.78,2.28)	1.06 (0.78,1.46)
Obese	5.03^*^ (1.19, 21.17)	0.75 (0.36,1.58)	0.57 (0.19,1.69)	1.38 (0.67,2.88)	1.19 (0.78,1.80)	4.55^*^ (1.04, 19.87)	0.83 (0.38,1.80)	0.58 (0.19,1.73)	1.29 (0.62,2.68)	1.15 (0.75,1.76)

^a^*multinomial logistic model;* ^b^ *binary logistic model;* ^c^ Confounding factors in the multiple regression analysis included age, sex, smoking, and TPOAb (only in multinomial logistic). Normal BMI group is used as reference. OR, odds ratio; 95%CI, 95% confidence interval. * Statistically significant

### TPOAb positivity

The results revealed that 736 (13.7%) participants were tested positive, and 4617 (86.3%) were tested negative for TPOAb (Table 1). The Obese group had a significantly higher prevalence of TPOAb positivity than the normal-weight group (17.3% vs. 11.6%, *P* < 0.001). Furthermore, the prevalence of TPOAb positivity in women was nearly twice that in men (17.1% vs. 9.1%, *P* < 0.05) (Table 1).

We observed a positive association between the odds of TPOAb positivity and BMI group; obese (OR: 1.59, 95% CI: 1.30–1.95, *P* < 0.05) and overweight (OR: 1.23, 95% CI: 1.02–1.47, *P* < 0.05) groups had increased odds of TPOAb positivity compared to the normal-BMI group. However, after adjustments for confounding factors, only the obese participants displayed a statistically significant positive association between the BMI group and the prevalence of TPOAb positivity (OR: 1.29, 95% CI: 1.04–1.60, *P* < 0.05) (Table 2). Unadjusted results from the sex-specific analysis showed that the odds of TPOAb positivity were positively associated with female overweight (OR: 1.26, 95% CI: 1.002–1.58, *P* < 0.05) and obese (OR: 1.51, 95% CI: 1.18–1.92, *P <* 0.05) BMI groups. However, after adjusting our results, only obese females showed a positive association with TPOAb positivity (OR: 1.37, 95% CI: 1.06–1.78, *P <* 0.05). No association was found between TPO positivity and different male BMI categories in our adjusted or unadjusted results. (Table 2). Moreover, we found a positive correlation between BMI and TPOAb level (r: 0.06, 95% CI: 0.03–0.08, *P <* 0.05); this positive correlation was also observed in women (r: 0.06, 95% CI: 0.03–0.10, *P* < 0.05), but not in men (Table 3).

**Table 3:** *Correlation of BMI with Thyroid Hormones and TPOAb*

	TSH (mIU/L)			FT4(ng/dL)			TPOAb (IU/ml)		
	Spearman correlation (CI)^a^	Crude % change (CI)^b^	Adjusted % change (CI)^c^	Spearman correlation (CI)^a^	Crude % change (CI)^b^	Adjusted % change (CI)^c^	Spearman correlation (CI)^a^	Crude % change (CI)^b^	Adjusted % change (CI)^c^
Total									
BMI (kg/m2)	0.04^*^ (0.01,0.07)	0.90^*^ (0.20,1.60)	0.82^*^ (0.11, 1.55)	−0.18^*^ (− 0.21,−0.16)	−0.69^*^ (− 0.81,−0.58)	−0.39^*^ (− 0.51, − 0.27)	0.06^*^ (0.03,0.08)	2.35^*^ (1.52, 3.19)	1.08^*^ (0.23, 1.95)
FEMALE									
BMI (kg/m2)	0.03 (− 0.01,0.06)	0.71 (− 0.25,1.67)	0.84 (− 0.17,1.86)	−0.16^*^ (− 0.19,−0.12)	−0.56^*^ (− 0.70,− 0.41)	-0.41^*^ (− 0.57,−0.26)	0.06^*^ (0.03,0,10)	2.45^*^ (1.32, 3.58)	1.62^*^ (0.42, 2.84)
MALE									
BMI (kg/m2)	0.01 (− 0.04,0.05)	0.33 (− 0.67,1.35)	0.62 (− 0.38,1.63)	−0.14^*^ (− 0.18,−0.10)	−0.60^*^ (− 0.79,−0.40)	− 0.44^*^ (− 0.63,−0.25)	0.01 (− 0.03,0.05)	0.58 (− 0.62, 1.79)	0.16 (− 1.04,1.37)

^a^ *95% CI based on 1000 bootstrap samples,* ^b^ *univariate linear regression model,* ^c^ multiple linear regression model. *Statistically significant. P-values less than 0.05 were considered statistically significant. Confounding factors in the multiple regression analysis included age, sex, and smoking (and TPOAb only for TSH and FT4 outcomes). Natural log transformations were used for outcomes; as a result, % changes were calculated as 100*(e^*coefficient*^*-1); CI, 95% confidence interval*

### Thyroid hormones and BMI association

We observed significantly higher TSH (1.69 (1.81) mU/L vs. 1.56 (1.68) mU/L, *P* < 0.001) and noticeable lower FT4 (1.17 (0.24) ng/dL vs. 1.25 (0.24) ng/dL, *P <* 0.001) serum levels in the obese group compared to the normal weight group (Table 1). In our linear regression analysis, BMI showed a positive and negative correlation with levels of serum TSH (r: 0.04, 95% CI: 0.01–0.07, *P <* 0.05) and FT4 (r: -0.18, 95% CI:  -0.21 – -0.16, *P <* 0.05) respectively. The sex-specific analysis showed that both men (r: -0.14, 95% CI:  -0.18 – -0.10, *P <* 0.05) and women (r: -0.16, 95% CI:  -0.19 – -0.12, *P <* 0.05) had a negative correlation between BMI and FT4, in contrast, neither men nor women showed a statistically significant correlation between TSH and BMI (Table 3).

## Discussion

The important results of this study are as follows: (1) the prevalence of hypothyroidism, both overt and subclinical, was higher in the obese group than the normal-weight group. (2) Obesity was associated with higher odds of overt hypothyroidism before and after adjustments for confounding factors; no association was observed between BMI categories and subclinical hypothyroidism. (3) Obesity was associated with increased odds of TPOAb positivity. (4) BMI was positively correlated with TSH and TPOAb serum levels and negatively correlated with serum FT4 levels.

Results regarding the association between obesity and thyroid function are still conflicting (Table 4). A recent meta-analysis performed by Song et al. using 22 studies found a positive association between obesity and risk of hypothyroidism (OR: 1.86; 95% CI: 1.63–2.11, *P* < 0.001); moreover, an increased odds of overt (OR: 3.21, 95% CI: 2.12–4.86, *P <* 0.001) and subclinical (OR: 1.70, 95% CI: 1.42–2.03, *P <* 0.001) hypothyroidism was observed in obese individuals. However, no association was found between obesity and hyperthyroidism [20]. Although most of our results are in agreement with this meta-analysis study, we found no association between subclinical hypothyroidism and obesity. A cross-sectional study conducted on 27,097 Norwegian individuals above 40 years found that obesity was positively associated with odds of subclinical and overt hypothyroidism. Furthermore, a positive correlation between BMI and TSH in both smoker and non-smoker participants was observed [28]. “The Blue Mountains Eye” cohort study conducted by Gopinath et al. on 1768 Australian individuals above 55 years old found a positive association between obesity and overt hypothyroidism; however, similar to our study, no significant association between obesity and prevalence of subclinical hypothyroidism was observed [22]. We observed a positive association between subclinical hyperthyroidism and the overweight group in our unadjusted results. However, this association was not seen in adjusted results or other BMI groups. Although a study by Holm et al. reported that obesity was associated with decreased risk of Graves’ hyperthyroidism [29], more studies are necessary to clarify the impact of obesity on hyperthyroidism.

**Table 4:** *Summary of Published Studies on the Association of Obesity with Thyroid Dysfunction*

Author, Year and Country	Study	Findings
Meta-analysis		
Song et al. 2019 [20]	Meta-analysis of 22 studies	-Positive association between obesity and overt hypothyroidism -Positive association between obesity and subclinical hypothyroidism -Positive association between obesity and TPOAb positivity
Cohort studies		
Knudsen et al. 2005 Denmark [21]	n: 4082, aged 18–65 years Average follow-up: 5 years	-Positive association between TSH and BMI -Positive association between FT4 and BMI
Gopinath et al., Australia 2010 [22]	n: 1768, age: ≥55 years Follow-up years: 5 years	-Positive association between obesity and overt hypothyroidism -No significant association between obesity and subclinical hypothyroidism
Soriguer et al. 2011 Spain [23]	F: 479/M: 305, age 18–65 years Follow-up: 6 years	-Obese participants had higher FT4 levels than control after fallow-up
Bjergved et al. Denmark 2014 [24]	F: 1577/M: 367, age 18–65 years Average follow-up: 11.2 years	-Positive association between BMI and TSH changes over follow-up -Negative association between FT4 change and BMI change only in women
Cross-sectional studies		
Manji et al. United Kingdom 2006 [25]	F: 361/M: 40, mean age 48.2 years	-No significant difference between obese and non-obese participants for TSH and FT4 levels
Rotondi et al. Italy 2009 [12]	F: 256/M: 94, mean age 46.2 ± 12.2 years	-Obese participants had lower FT4 and higher TSH levels, no correlation was seen between TSH and FT4 with BMI
Ambrosi et al. Italy 2010 [13]	Only overweight and obese individuals, F: 436/M: 145, mean age: 39.8 ± 13.7 years	-TSH was positively correlated with BMI
Sakurai et al. Japan 2014 [26]	F: 993/M: 1044, age 36–55 years	-Positive association between BMI and TSH only in men
Bétry et al. France 2015 [8]	Only included obese participants, F: 554/M: 246, mean age: 44 ± 0.5	-BMI and leptin were positively associated with TSH
Al-Musa et al. Saudi Arabia 2017 [16]	n:278, F: 48.2% M: 51.8%	-Positive correlation between BMI and TSH -BMI had no statistically significant correlation with FT4
Valdés et al., 2017 Spain [27]	n: 3928, age: 18–93 F: 54% M: 46%	-Positive association between BMI and TSH levels

*F* Female, *M* Male, *n* Number of participants

We found that BMI had a positive correlation with TSH and TPOAb and a negative correlation with FT4 levels. A cohort study performed by Knudsen et al. in Denmark with 4082 participants aged 18–65 and an average follow-up period of 5 years found that serum TSH was positively associated with BMI. Furthermore, lower levels of FT4 were associated with higher BMI [21]. Valdés et al. reported a positive correlation between TSH and BMI from a cross-sectional study performed on 3928 individuals in Spain [27]. Soriguer et al. reported a controversial finding in a cohort study on 784 individuals in Spain; in contrast to most studies and our study, this study reported a positive association between BMI and FT4 after a six-year follow-up [23]. A cross-sectional study performed by Manji et al. on 401 euthyroid individuals in Britain found no difference between obese and lean participants regarding TSH and FT4 levels; additionally, no association was found between BMI and TSH or FT4 [25]. Another cross-sectional study conducted by Shinkov et al. on 2153 participants in Bulgaria found no correlation between TSH and BMI [30].

Many mechanisms have been proposed to explain the observed association between obesity and thyroid dysfunction. Several studies have found a positive correlation between increased serum leptin and TSH levels in obese individuals [8, 31]. Leptin promotes TRH expression and synthesis in the paraventricular hypothalamic and arcuate nucleus, which, in turn, can cause an increase in serum TSH levels [32]. Leptin could also increase the conversion of T4 to T3 in peripheral tissues resulting in decreased levels of T4 that is observed in many studies. Moreover, this can lead to compensatory activation of the hypothalamus-pituitary-thyroid axis in an attempt to maintain serum thyroid hormone levels within the euthyroid range [33, 34]. Adipocytes of obese individuals generate a state of peripheral resistance to thyroid hormones due to their lower-than-normal expression of TSH receptors, leading to increased plasma TSH levels as a compensatory mechanism [35]. Although it is not possible to precisely assert whether thyroid function changes are primary or secondary to obesity, several studies suggest obesity to be the primary factor that triggers thyroid function changes. This conclusion is based on the reversion or alleviation of thyroid dysfunction after weight loss in obese individuals [35, 36]. Indeed, weight loss is proposed to be a key factor in the restoration of hormonal imbalances observed in obesity [37]. It is likely that both states, thyroid dysfunction primary or secondary to obesity, to some degree, are involved in all studies.

Regarding the relation between TPOAb and obesity, there are inconsistencies among studies. Although many studies have found a positive association between obesity and TPOAb [20, 38], some have found no association linking TPOAb and obesity [21, 25]. Hence, more studies are needed to illuminate the relationship between TPOAb and obesity.

Adipokines and inflammatory cytokines released by the adipose tissue in obese individuals might play a role in altering the thyroid function by inducing chronic low-grade inflammation in the thyroid tissue of obese individuals [20, 39]. Moreover, several studies have shown that leptin can decrease the function of regulatory T (Treg) cells and increase the percentage of T helper 1 (Th1) cells; these events can, to some extent, explain the reported positive correlation between obesity and TPOAb positivity by stating that as a result of altered immune function, obese individuals are more prone to autoimmune and inflammatory processes in the thyroid gland [40, 41].

Sex-specific results of this study showed a positive association between male and female obese groups and overt hypothyroidism. Several theories have been proposed to explain the different effects of obesity on male and female individuals’ thyroid function. One theory suggests that different body fat distribution in men and women could result in distinct peripheral metabolism of thyroid hormones leading to different obesity-related changes in thyroid hormone levels. Furthermore, since various sex hormones such as estradiol and testosterone have diverse effects on thyroid function, differences in serum sex hormones between men and women could play a role in different obesity-induced thyroid dysfunction patterns observed among men and women [42–44].

One point should be noted for the interpretation of our results. The association between overweight and the odds of overt hypothyroidism was no longer significant after adjusting for confounding factors, such as sex and age. Furthermore, in women, when the results were adjusted, obesity and the risk of overt hypothyroidism were not significantly associated. Since it is known that the female gender [45] and older age [46] are positively associated with hypothyroidism and obesity, the results of this study should be interpreted with caution.

The findings of this study should be seen in the light of some limitations. While the cross-sectional study design is suitable for establishing prevalence, determination of the observed associations’ causality and temporality are not possible. Even though the results were adjusted for several confounding factors, as with all observational studies, the presence of additional unaccounted confounding factors might have affected the results. Moreover, this study does not take into account the common variability of thyroid function tests in an individual; consequently, there is a possibility that some individuals with temporary thyroid function alterations might have been misclassified due to the cross-sectional nature of the survey. The exclusion criteria did not include the usage of all drugs with potential effects on thyroid function. Obesity and overweight were defined solely based on BMI values. Thyroid examination and sonography were not fully documented for all participants. Finally, the analyses did not include other thyroid-related serum elements such as Free triiodothyronine (FT3) and Anti-TSH receptor antibodies.

This study had several merits. In contrast to many studies on this subject, our study had a large community-based sample, which increased statistical power and accuracy. Furthermore, the population-specific TSH and FT4 reference ranges of this study were based on results obtained from a previous survey of TTS participants to minimize misclassification errors. BMI was utilized as both a categorical and a continuous variable to clarify the statistical association between BMI and thyroid function changes. Additionally, sex-specific subgroups were used to further investigate the effects of elevated BMI on thyroid function in different genders.

## Conclusion

In conclusion, this study found a higher prevalence of subclinical and overt hypothyroidism among Iranian adult obese individuals. Moreover, obesity was positively associated with increased odds of overt hypothyroidism and TPOAb positivity in obese participants even after adjustments for confounding factors. However, no association was found between subclinical hypothyroidism and obesity. Furthermore, BMI was positively correlated with serum TSH and TPOAb and negatively correlated with serum FT4 levels.

Even though several theories have been proposed to explain the complex relationship between obesity and thyroid dysfunction, the exact physiological nature of the association between obesity and thyroid dysfunction is still unclear. Consequently, further researches are needed to clarify this relationship. More studies are also required to uncover the exact mechanism which causes the impact of obesity on thyroid function to be different among men and women. Furthermore, Even though obesity is associated with elevated TSH, some studies suggest that isolated, moderately elevated TSH levels in an obese individual, in the absence of autoimmunity and hypothyroidism signs, could be regarded as normal [12, 27]. However, further researches are needed to test the accuracy of this statement.

## Data Availability

The data that support the findings of this study are available from the Research Institute for Endocrine Sciences at Shahid Beheshti University of Medical Sciences, but restrictions apply to the availability of these data due to Iranian data sharing policies on surveyed epidemiological data. However, data are available from the corresponding author upon reasonable request and after completion of a data-sharing agreement and approval by the ethics committee of the Research Institute for Endocrine Sciences. Upon completion of the aforementioned steps, data sharing will be conducted in a safe environment under the regulations and supervision of the IT office of Shahid Beheshti University of Medical Sciences.

## References

[1] GBD 2015 Obesity Collaborators. Health effects of overweight and obesity in 195 countries over 25 years. N Engl J Med. 2017;377(1):13–27. 10.1056/NEJMoa1614362 PMC5477817 28604169

[2] Blüher M. Obesity: global epidemiology and pathogenesis. Nat Rev Endocrinol. 2019;15(5):288–98. 10.1038/s41574-019-0176-8 30814686

[3] Rahmani A, Sayehmiri K, Asadollahi K, Sarokhani D, Islami F, Sarokhani M. Investigation of the prevalence of obesity in Iran: a systematic review and meta-analysis study. Acta Med Iran. 2015:596–607. 26615371

[4] Tabrizi JS, Sadeghi-Bazargani H, Farahbakhsh M, Nikniaz L, Nikniaz Z. Prevalence and associated factors of overweight or obesity and abdominal obesity in Iranian population: a population-based study of northwestern Iran. Iran J Public Health. 2018;47(10):1583. PMC6277719 30524990

[5] Sajjadi-Jazi SM, Sharifi F, Varmaghani M, Meybodi HA, Farzadfar F, Larijani B. Epidemiology of hyperthyroidism in Iran: a systematic review and meta-analysis. J Diabetes Metab Disord. 2018;17(2):345–55. 10.1007/s40200-018-0367-1 PMC6405392 30918870

[6] Amouzegar A, Mehran L, Takyar M, Abdi H, Azizi F. Tehran thyroid study (TTS). Int J Endocrinol Metab. 2018;16(4 Suppl). 10.5812/ijem.84727 PMC6289306 30584429

[7] Ortiz VE, Kwo J. Obesity: physiologic changes and implications for preoperative management. BMC Anesthesiol. 2015;15(1):1–2. 10.1186/s12871-015-0079-8 PMC4491231 26141622

[8] Betry C, Challan-Belval MA, Bernard A, Charrie A, Drai J, Laville M, Thivolet C, Disse E. Increased TSH in obesity: Evidence for a BMI-independent association with leptin. Diab Metab. 2015;41(3):248–51. 10.1016/j.diabet.2014.11.009 25541439

[9] Zhang X, Li Y, Zhou X, Han X, Gao Y, Ji L (2019). Association between serum thyrotropin within the euthyroid range and obesity. Endocr J.

[10] An YM, Moon SJ, Kim SK, Suh YJ, Lee JE. Thyroid function in obese Korean children and adolescents: Korea National Health and Nutrition Examination Survey 2013–2015. Ann Pediatr Endocrinol Metab. 2018;23(3):141. 10.6065/apem.2018.23.3.141 PMC6177661 30286570

[11] Muscogiuri G, Sorice GP, Mezza T, Prioletta A, Lassandro AP, Pirronti T, Della Casa S, Pontecorvi A, Giaccari A. High normal tsh values in obesity: Is it insulin resistance or adipose tissue's guilt?. Obesity. 2013;21(1):101–6. 10.1002/oby.20240 23505173

[12] Rotondi M, Leporati P, La Manna A, Pirali B, Mondello T, Fonte R, Magri F, Chiovato L. Raised serum TSH levels in patients with morbid obesity: is it enough to diagnose subclinical hypothyroidism?. Eur J Endocrinol. 2009;160(3):403. 10.1530/EJE-08-0734 19073832

[13] Ambrosi B, Masserini B, Iorio L, Delnevo A, Malavazos AE, Morricone L, Sburlati LF, Orsi E (2010). Relationship of thyroid function with body mass index and insulin-resistance in euthyroid obese subjects. J Endocrinol Investig.

[14] Ren R, Jiang X, Zhang X, Guan Q, Yu C, Li Y, Gao L, Zhang H, Zhao J (2014). Association between thyroid hormones and body fat in euthyroid subjects. Clin Endocrinol.

[15] Lambrinoudaki I, Armeni E, Rizos D, Georgiopoulos G, Athanasouli F, Triantafyllou N, Panoulis K, Augoulea A, Creatsa M, Alexandrou A, Alevizaki M, Stamatelopoulos K (2015). Indices of adiposity and thyroid hormones in euthyroid postmenopausal women. Eur J Endocrinol.

[16] Al-Musa HM. Impact of obesity on serum levels of thyroid hormones among euthyroid Saudi adults. J Thyroid Res. 2017;2017. 10.1155/2017/5739806. 10.1155/2017/5739806 PMC5463130 28630779

[17] Azizi F, Ghanbarian A, Momenan AA, Hadaegh F, Mirmiran P, Hedayati M, Mehrabi Y, Zahedi-Asl S. Prevention of non-communicable disease in a population in nutrition transition: Tehran Lipid and Glucose Study phase II. Trials. 2009;10(1):1–5. 10.1186/1745-6215-10-5 PMC2656492 19166627

[18] Amouzegar A, Delshad H, Mehran L, Tohidi M, Khafaji F, Azizi F. Reference limit of thyrotropin (TSH) and free thyroxine (FT 4) in thyroperoxidase positive and negative subjects: a population based study. J Endocrinol Investig. 2013;36(11):950–4. 10.3275/9033 23873252

[19] AZIZI F, Hadaegh F, KHALILI D, Esteghamati A, HOSSEIN PF, Delavari A (2010). Appropriate definition of metabolic syndrome among Iranian adults: report of the Iranian National Committee of obesity.

[20] Song RH, Wang B, Yao QM, Li Q, Jia X, Zhang JA. The impact of obesity on thyroid autoimmunity and dysfunction: a systematic review and meta-analysis. Front Immunol. 2019;10:2349. 10.3389/fimmu.2019.02349 PMC6797838 31681268

[21] Knudsen N, Laurberg P, Rasmussen LB, Bülow I, Perrild H, Ovesen L, Jørgensen T. Small differences in thyroid function may be important for body mass index and the occurrence of obesity in the population. J Clin Endocrinol Metab. 2005;90(7):4019–24. 10.1210/jc.2004-2225 15870128

[22] Gopinath B, Wang JJ, Kifley A, Wall JR, Eastman CJ, Leeder SR, Mitchell P. Five‐year incidence and progression of thyroid dysfunction in an older population. Intern Med J. 2010;40(9):642–9. 10.1111/j.1445-5994.2009.02156.x 20840213

[23] Soriguer F, Valdes S, Morcillo S, Esteva I, Almaraz MC, de Adana MS, Tapia MJ, Dominguez M, Gutierrez‐Repiso C, Rubio‐Martin E, Garrido‐Sanchez L. Thyroid hormone levels predict the change in body weight: a prospective study. Eur J Clin Investig. 2011;41(11):1202–9. 10.1111/j.1365-2362.2011.02526.x 21470220

[24] Bjergved L, Jørgensen T, Perrild H, Laurberg P, Krejbjerg A, Ovesen L, Rasmussen LB, Knudsen N. Thyroid function and body weight: a community-based longitudinal study. PLoS One. 2014;9(4):e93515. 10.1371/journal.pone.0093515 PMC3984087 24728291

[25] Manji N, Boelaert K, Sheppard MC, Holder RL, Gough SC, Franklyn JA. Lack of association between serum TSH or free T4 and body mass index in euthyroid subjects. Clin Endocrinol. 2006;64(2):125–8. 10.1111/j.1365-2265.2006.02433.x 16430708

[26] Sakurai M, Nakamura K, Miura K, Yoshita K, Takamura T, Nagasawa SY, Morikawa Y, Ishizaki M, Kido T, Naruse Y, Nakashima M. Association between a serum thyroid-stimulating hormone concentration within the normal range and indices of obesity in Japanese men and women. Intern Med. 2014;53(7):669–74. 10.2169/internalmedicine.53.1387 24694474

[27] Valdés S, Maldonado Araque C, Lago Sampedro A, Lillo Muñoz JA, Garcia Fuentes E, Perez Valero V, Gutiérrez Repiso C, Garcia Escobar E, Goday A, Urrutia I, Peláez L. Reference values for TSH may be inadequate to define hypothyroidism in persons with morbid obesity: Di@ bet. es study. Obesity. 2017;25(4):788–93. 10.1002/oby.21796 28276648

[28] Asvold BO, Bjoro T, Vatten LJ (2009). Association of serum TSH with high body mass differs between smokers and never-smokers. J Clin Endocrinol Metab.

[29] Holm IA, Manson JE, Michels KB, Alexander EK, Willett WC, Utiger RD (2005). Smoking and other lifestyle factors and the risk of Graves’ hyperthyroidism. Arch Intern Med.

[30] Shinkov A, Borissova AM, Kovatcheva R, Atanassova I, Vlahov J, Dakovska L. The prevalence of the metabolic syndrome increases through the quartiles of thyroid stimulating hormone in a population-based sample of euthyroid subjects. Arq Bras Endocrinol Metabologia. 2014;58(9):926–32. 10.1590/0004-2730000003538 25627048

[31] Iacobellis G, Cristina Ribaudo M, Zappaterreno A, Valeria Iannucci C, Leonetti F. Relationship of thyroid function with body mass index, leptin, insulin sensitivity and adiponectin in euthyroid obese women. Clin Endocrinol. 2005;62(4):487–91. 10.1111/j.1365-2265.2005.02247.x 15807881

[32] Ghamari-Langroudi M, Vella KR, Srisai D, Sugrue ML, Hollenberg AN, Cone RD. Regulation of thyrotropin-releasing hormone-expressing neurons in paraventricular nucleus of the hypothalamus by signals of adiposity. Mol Endocrinol. 2010;24(12):2366–81. 10.1210/me.2010-0203 PMC2999480 20943814

[33] Kumar HK, Yadav RK, Prajapati J, Reddy CV, Raghunath M, Modi KD. Association between thyroid hormones, insulin resistance, and metabolic syndrome. Saudi Med J. 2009;30(7):907–11. 19618005

[34] Ortega FJ, Jilkova ZM, Moreno-Navarrete JM, Pavelka S, Rodriguez-Hermosa JI, Kopeckỳ J, Fernandez-Real JM. Type I iodothyronine 5′-deiodinase mRNA and activity is increased in adipose tissue of obese subjects. Int J Obes. 2012;36(2):320–4. 10.1038/ijo.2011.101 21610697

[35] Nannipieri M, Cecchetti F, Anselmino M, Camastra S, Niccolini P, Lamacchia M, Rossi M, Iervasi G, Ferrannini E. Expression of thyrotropin and thyroid hormone receptors in adipose tissue of patients with morbid obesity and/or type 2 diabetes: effects of weight loss. Int J Obes. 2009;33(9):1001–6. 10.1038/ijo.2009.140 19636322

[36] Lips MA, Pijl H, van Klinken JB, de Groot GH, Janssen IM, Van Ramshorst B, Van Wagensveld BA, Swank DJ, Van Dielen F, Smit JW. Roux-en-Y gastric bypass and calorie restriction induce comparable time-dependent effects on thyroid hormone function tests in obese female subjects. Eur J Endocrinol. 2013;169(3):339–47. 10.1530/EJE-13-0339 23811187

[37] Pasquali R, Casanueva F, Haluzik M, Van Hulsteijn L, Ledoux S, Monteiro MP, Salvador J, Santini F, Toplak H, Dekkers OM. European Society of Endocrinology Clinical Practice Guideline: endocrine work-up in obesity. European J Endocrinol. 2020;182(1):G1–32. 10.1530/EJE-19-0893 31855556

[38] Marzullo P, Minocci A, Tagliaferri MA, Guzzaloni G, Di Blasio A, De Medici C, Aimaretti G, Liuzzi A. Investigations of thyroid hormones and antibodies in obesity: leptin levels are associated with thyroid autoimmunity independent of bioanthropometric, hormonal, and weight-related determinants. J Clin Endocrinol Metab. 2010;95(8):3965–72. 10.1210/jc.2009-2798 20534769

[39] García-García E, Vázquez-López MA, García-Fuentes E, Galera-Martínez R, Gutiérrez-Repiso C, García-Escobar I, Bonillo-Perales A. Thyroid function and thyroid autoimmunity in relation to weight status and cardiovascular risk factors in children and adolescents: a population-based study. J Clin Res Pediatr Endocrinol. 2016;8(2):157. 10.4274/jcrpe.2687 PMC5096470 26761948

[40] Procaccini C, Carbone F, Galgani M, La Rocca C, De Rosa V, Cassano S, Matarese G. Obesity and susceptibility to autoimmune diseases. Expert Rev Clin Immunol. 2011;7(3):287–94. 10.1586/eci.11.18 21595595

[41] Fresno M, Alvarez R, Cuesta N. Toll-like receptors, inflammation, metabolism and obesity. Arch Physiol Biochem. 2011;117(3):151–64. 10.3109/13813455.2011.562514 21599616

[42] Chen Y, Chen Y, Xia F, Wang N, Chen C, Nie X, Li Q, Han B, Zhai H, Jiang B, Shen Z. A higher ratio of estradiol to testosterone is associated with autoimmune thyroid disease in males. Thyroid. 2017;27(7):960–6. 10.1089/thy.2016.0661 28558486

[43] Laurberg P, Knudsen N, Andersen S, Carlé A, Pedersen IB, Karmisholt J. Thyroid function and obesity. Eur Thyroid J. 2012;1(3):159–67. 10.1159/000342994 PMC3821486 24783015

[44] Saltevo J, Kautiainen H, Vanhala M. Gender differences in adiponectin and low-grade inflammation among individuals with normal glucose tolerance, prediabetes, and type 2 diabetes. Gend Med. 2009;6(3):463–70. 10.1016/j.genm.2009.09.006 19850242

[45] Dunn D, Turner C. Hypothyroidism in women. Nurs Women's Health. 2016;20(1):93–8. 10.1016/j.nwh.2015.12.002 26902444

[46] Leng O, Razvi S. Hypothyroidism in the older population. Thyroid Res. 2019;12(1):1–0. 10.1186/s13044-019-0063-3 PMC6367787 30774717

